# Intimate partner violence against adolescents and young women in sub-Saharan Africa: who is most vulnerable?

**DOI:** 10.1186/s12978-021-01077-z

**Published:** 2021-06-17

**Authors:** Yohannes Dibaba Wado, Martin K. Mutua, Abdu Mohiddin, Macellina Y. Ijadunola, Cheikh Faye, Carolina V. N. Coll, Aluisio J. D. Barros, Caroline W. Kabiru

**Affiliations:** 1 African Population and Health Research Center, Nairobi, Kenya; 2 Aga Khan University, Nairobi, Kenya; 3 Department of Epidemiology and Medical Statistics, University of Ibadan, Ibadan, Nigeria; 4 International Centre for Equity in Health, Universidade Federal de Pelotas, Pelotas, RS Brazil

**Keywords:** Physical or sexual violence, Attitudes, SSA, Adolescents and young women, Equiplot

## Abstract

**Background:**

Intimate partner violence (IPV) is a global public health and human rights issue that affects millions of women and girls. While disaggregated national statistics are crucial to assess inequalities, little evidence exists on inequalities in exposure to violence against adolescents and young women (AYW). The aim of this study was to determine inequalities in physical or sexual IPV against AYW and beliefs about gender based violence (GBV) in sub-Saharan Africa (SSA).

**Methods:**

We used data from the most recent Demographic and Health Surveys (DHS) conducted in 27 countries in SSA. Only data from surveys conducted after 2010 were included. Our analysis focused on married or cohabiting AYW aged 15–24 years and compared inequalities in physical or sexual IPV by place of residence, education and wealth. We also examined IPV variations by AYW’s beliefs about GBV and the association of country characteristics such as gender inequality with IPV prevalence.

**Results:**

The proportion of AYW reporting IPV in the year before the survey ranged from 6.5% in Comoros to 43.3% in Gabon, with a median of 25.2%. Overall, reported IPV levels were higher in countries in the Central Africa region than other sub-regions. Although the prevalence of IPV varied by place of residence, education and wealth, there was no clear pattern of inequalities. In many countries with high prevalence of IPV, a higher proportion of AYW from rural areas, with lower education and from the poorest wealth quintile reported IPV. In almost all countries, a greater proportion of AYW who approved wife beating for any reason reported IPV compared to their counterparts who disapproved wife beating. Reporting of IPV was weakly correlated with the Gender Inequality Index and other societal level variables but was moderately positively correlated with adult alcohol consumption (r = 0.48) and negative attitudes towards GBV (r = 0.38).

**Conclusion:**

IPV is pervasive among AYW, with substantial variation across and within countries reflecting the role of contextual and structural factors in shaping the vulnerability to IPV. The lack of consistent patterns of inequalities by the stratifiers within countries shows that IPV against women and girls cuts across socio-economic boundaries suggesting the need for comprehensive and multi-sectoral approaches to preventing and responding to IPV.

## Plain English summary

Intimate partner violence (IPV) is a global public health and human rights issue that affects millions of women and girls. Disaggregated national data are needed to assess inequalities in exposure to violence against adolescents and young women (AYW).

In this study, we examined inequalities in physical or sexual IPV against AYW in sub-Saharan Africa (SSA) using data from the most recent national surveys from 27 countries. Our analyses focused on married or cohabiting adolescents and young women aged 15–24 years and compared inequalities in IPV by place of residence, education and wealth. We also examined IPV variations by AYW’s beliefs and country characteristics such as gender inequality.

The percentage of AYW reporting IPV in the year before the survey ranged from 6.5% in Comoros to 43.3% in Gabon. Overall, IPV levels were higher in countries in the Central Africa region than other sub-regions. IPV levels varied by place of residence, education and wealth although there was no clear pattern of inequalities. A higher percentage of AYW from rural areas, with lower education and from the poorest households reported IPV. In almost all countries, a higher percentage of AYW who approved wife beating reported IPV compared to their counterparts who disapproved wife beating.

IPV is pervasive with substantial variations between and within countries reflecting the role of contextual and structural factors in shaping the vulnerability to IPV. There is a need for comprehensive and multi-sectoral approaches to preventing and responding to IPV against AYW.

## Background

Violence against women and girls is a global public health and human rights issue that affects millions of women and girls. According to the World Health Organization approximately one-third of women globally have experienced some form of violence (physical or sexual) from a partner or non-partner in their lifetime [1]. While the effects of gender based violence (GBV) on the physical, mental health and social well-being of women and girls are relatively well-documented [2–4], its health consequences continue to be unabated due to the persistent high prevalence. For instance, young women in sub-Saharan Africa (SSA) continue to carry the brunt of high HIV infection due to sexual violence, poverty and social norms around marriage, gender inequalities and harmful traditional practices that reinforce unequal power dynamics with young women particularly disadvantaged [5–8].

Gender inequalities increase the risk of violence against women and girls and inhibit the ability of those affected to seek protection [9, 10]. Adolescent girls and young women (AYW), particularly those married to older men, and/or married as children or adolescents, may be disproportionately at risk of being exploited and violated because they have less bargaining power within their relationships [10, 11]. Data from the WHO violence against women surveys show that globally 30% of adolescent girls (aged 15 to 19 years) have experienced physical and/or sexual violence by an intimate partner in their lifetime [3]. Moreover, one study that used Demographic and Health Survey (DHS) data from 30 developing countries estimated that 28% of adolescents (15–19 years) and 29% of young women (20–24 years) had experienced physical or sexual intimate partner violence (IPV) [12].

Much research on the determinants of IPV against women has been informed by an ecological framework that outlines multiple factors operating at different levels—individual, relationship, community, and societal levels—that explain why some groups of people are at greater risk [13–15]. For instance, socioeconomic inequalities and socio-cultural norms such as those around male dominance over women contribute to the high prevalence of GBV in SSA [12, 16]. Evidence from a systematic review also shows that individual characteristics such as age, age difference with the partner, and education level are risk factors of GBV [17]. Moreover, there are various contextual and country-specific drivers of violence in SSA. There is a strong link between poverty and violence among young women with those from poor households and communities being at greater risk [18]. Low education, exposure to violence in childhood, unequal power in intimate relationships, and attitudes and norms accepting violence and gender inequality also increase the risk of experiencing IPV and sexual violence [13].

With the Sustainable Development Goals (SDGs), the international community committed to the achievement of gender equality and elimination of all forms of violence against women and girls by 2030. Promoting gender equality, preventing violence against women and girls (SDG, goal 5) and ensuring responsive and inclusive societies (SDG target 16.1) are far-reaching goals in the SDGs to ensure gender equity [19]. While the relationship between gender and violence is complex, evidence indicates that gender inequalities increase the risk of violence by men against women and inhibit the ability of those affected to seek protection [9, 20]. SDG target 17.18 also calls for disaggregated national statistics by income, rural–urban residence, gender and other variables to assess inequalities. Yet, little evidence exists on inequalities in exposure to violence against AYW in SSA. In this regard, the agenda to “leave no one behind” and Countdown to 2030 are well-timed to provide inequality data for the purpose of designing effective interventions to improved gender equity and address violence against AYW in SSA countries [21] since existing evidence of interventions are skewed towards high income countries [20, 22, 23].

Demographic and Health Surveys are an important source of data to study cross-national and regional inequalities in exposure to IPV because they are nationally-representative and use standardized tools that follow ethical and safety recommendations for research on domestic violence against women [24]. Such cross-national and regional comparisons will enable the identification of groups of AYW that are most affected. As the DHS are conducted about every 5 years, they are valuable in monitoring the progress and effectiveness of interventions targeting the protection and empowerment of AYW to prevent violence. Population-based surveys that highlight differences in IPV by wealth index, residence and education and other individual- and community-level determinants of violence against AYW are useful for informing the design and targeting of interventions. In this study, we drew on DHS data to examine inequalities in IPV against AYW in SSA and identify groups that experience the highest levels of violence in different contexts.

## Methods

### Data

We used data from the most recent DHS with the violence against women module. We limited our analyses to data from 27 countries in SSA whose most recent survey was conducted after 2010. Eight countries with surveys were excluded because the violence module was not applied (Guinea, Lesotho, Madagascar, Mauritania and Guinea) or only had data from national surveys carried out before 2010 (Ghana, Liberia and Sao Tome). We also included similar number of countries with at least one round of surveys since 2010 for the analysis of AYW’s attitudes towards wife beating. The study population included married or cohabiting adolescent girls aged 15–19 years and young women aged 20–24 years. We used the United Nations Population Division grouping of countries in SSA into four sub-regions: Central Africa, Eastern Africa, Southern Africa and Western Africa. Table 1 presents the countries included in the study by region and year of survey.

**Table 1:** List of countries by year of DHS surveys for the intimate partner violence analysis

	Region	Country	IPV data	Most recent survey
Eastern and Southern Africa	Eastern Africa	Rwanda	2014	2014
		Malawi	2015	2015
		Kenya	2014	2014
		Ethiopia	2016	2016
		Zambia	2013	2013
		Burundi	2016	2016
		Tanzania	2015	2015
		Uganda	2016	2016
		Mozambique	2015	2015
		Comoros	2012	2012
	Southern Africa	Zimbabwe	2016	2016
		Namibia	2013	2013
		South Africa	2016	2016
West and Central Africa	West Africa	Mali	2018	2018
		Senegal	2017	2017
		Burkina Faso	2010	2010
		Sierra Leone	2013	2013
		Nigeria	2018	2018
		Togo	2013	2013
		Cote d’ivoire	2011	2011
		Benin	2017	2017
		Gambia	2013	2013
	Central Africa	Congo Brazzaville		2011
		Gabon	2012	2012
		Cameroon	2011	2011
		Angola	2015	2015
		Congo DRC	2013	2013
		Chad	2014	2014

The equity stratifiers used in this analysis included household wealth quintiles, rural–urban residence, woman’s education, age and age difference with the partner. Accordingly, inequality in this analysis refers to differences in the outcome indicator (IPV against AYW) between two or more sub-groups. In the DHS, the wealth index is coded into five quantiles, however, in our analyses, we compared the two extreme categories (the first quintile vs. the fifth quintile). The first quintile represents the poorest 20% in the population and the fifth quintile represents the wealthiest 20% [25]. Education was coded into two categories (primary or less, and secondary or more) based on the distribution of AYW’s education. In the majority of the countries included in this analysis, only a small proportion of AYW had no formal education. Age was recoded into two categories: adolescents (15–19 years) and young women (age 20–24 years). Age difference with the partner was categorized into two groups based on the distribution of the data (a difference of more than 5 years vs. a difference of 5 years or less).

The main outcome variable was intimate partner violence (physical, sexual and physical or sexual violence) against AYW in the past year. The current prevalence of IPV was defined as the percentage of currently married or cohabiting AYW who reported having experienced at least one act of IPV in the 12 months before the survey. In the DHS, violence information is obtained from ever-married and cohabiting respondents on violence committed by their current and former spouses/partners and by others. Physical IPV is measured using a shortened, modified version of the Conflict Tactics Scale [26] which asks the respondent if she has ever been—pushed, shaken, slapped, punched with a fist or something that could hurt, kicked, dragged or beaten up, choked, burned on purpose or had something thrown at her [27]. Sexual violence was assessed using the following items: physically forced to have unwanted sex, or forced into other unwanted sexual acts. An affirmative answer to one or more of the items listed in the Conflict Tactics Scale constitutes evidence of physical and sexual violence (see DHS reports, www.dhsprogram.org). We also looked at the prevalence of a combination of the two types of violence (physical or sexual).

The DHS collects various proxy indicators of women’s empowerment including attitudes towards wife beating, also named beliefs towards gender based violence. As attitudes towards violence is one of the key predictors of exposure to GBV [15, 28], we examine variation in attitudes towards wife beating by the stratifiers. In the DHS, all respondents are asked a series of questions to assess their attitudes to wife-beating. The questions ask whether a husband is justified in hitting or beating his wife in any of the following scenarios; if she goes out without telling him; if she neglects the children; if she argues with him; if she refuses to have sex with him and if she burns the food. A single composite variable ‘disagreement with wife-beating’ was constructed by grouping women into two categories: women who endorse at least one reason for wife-beating and women who reject all reasons of wife-beating. Unlike the IPV, which is administered to a sub-sample of households, the attitudes towards violence data is collected from all women.

In addition, we performed an ecological analysis and used multiple data sets to examine the correlation between country IPV prevalence with key societal characteristic: Gross National Income per capita extracted from the World Bank [29]; Gender Inequality Index (GII) an index that measures gender inequalities in reproductive health, empowerment and labor force participation, from the UNDP [30]; Fragile States Index—a measure of state fragility and instability—extracted from the 2019 Fragile States Index annual report [31]; urbanization levels from PRB’s world population data sheet [32]; educational attainment and AYW’s beliefs about GBV both extracted from the DHS; and adult male alcohol consumption per capita extracted from the WHO data base [33].

### Data analysis

Data were analyzed using STATA software version 14 (StataCorp, 2015). We used proportions to estimate prevalence of IPV at country level by the stratifiers. The median and the interquartile range were used to summarize IPV prevalence at regional and sub-regional levels. We analyzed survey data for each country at the national level, to compare the prevalence of IPV (physical or sexual violence) among AYW by the equity stratifiers—wealth quintiles, education, place of residence, and age. We also examined how attitudes towards wife beating, one of the major predictors of IPV, vary by the stratifiers. Data presented are weighted using the domestic violence weight, to adjust for within country sample selection and nonresponse. We use equiplots and charts to visualize inequalities in IPV against AYW by the stratifiers. Equiplots are used to present intervention coverage by groups, making it possible to visualize both the level of coverage in each group and the distance between groups, which represents absolute inequality [34]. We also use other relative measures of inequality such as ratios to demonstrate how one sub-group differs from the other. Moreover, proportions of key outcome indicators are presented with their confidence intervals in tables. Accordingly, significant differences between sub-groups are determined based on non-overlapping confidence intervals. To assess the association of IPV with selected societal determinants (at the country level) we used scatter plots, Pearson correlation coefficient and linear regression analyses to test associations with IPV prevalence at the 5% level. For some of the indicators, we did log transformations to reduce the influence of outliers.

## Results

### Violence against adolescents and young women

**Fig. 1:**
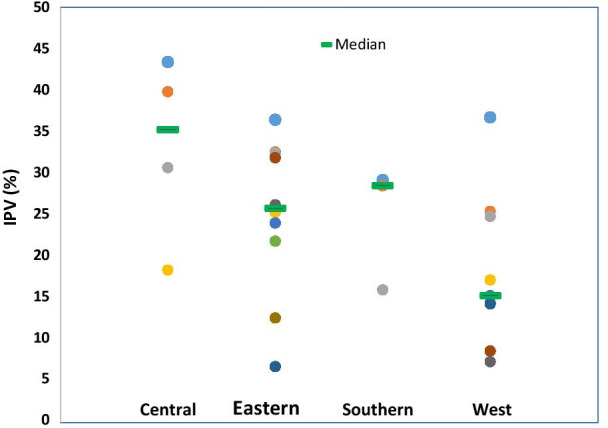
Median prevalence of physical or sexual intimate partner violence against AYW by region, SSA

Across the 27 countries, the reported prevalence of physical or sexual IPV ranged from 6.5% in Comoros (2012) to 43.3% in Gabon (2012). It varied widely between the countries. The median prevalence of combined physical or sexual IPV against AYW was 25.2%. The prevalence was highest in Central Africa (39.8%), followed by Southern Africa (28.4%) respectively (Fig. 1; Additional file 1: Table S1). In 5 of the 27 countries (Gabon, Burundi, DRC Congo, Cameroon, and Sierra Leone) more than 35% of AYW reported experiencing either physical or sexual IPV in the 12 months before the survey. Within the sub-regions, large inequalities between countries were observed in Eastern Africa. In East Africa, the prevalence of physical or sexual IPV varied from 36.4% in Burundi to only 6.5% in Comoros (Table 2).

**Table 2:** Percentage of adolescents and young women 15–24 years who experienced intimate partner violence, national DHS surveys, 2010–2018 (in parenthesis 95% confidence interval)

Country	Physical and or sexual IPV	Physical IPV	Sexual IPV	N
Gabon	43.4 [36.7,50.3]	38.8 [32.3,45.6]	16.1 [11.6,21.9]	573
Congo DRC	40.9 [36.8,45.1]	32.6 [29.1,36.4]	23.9 [20.0,28.3]	1136
Cameroon	39.8 [36.1,43.5]	33.4 [29.7,37.4]	16.3 [13.6,19.5]	943
Sierra Leone	36.7 [32.2,41.4]	32.9 [28.7,37.4]	8.6 [6.3,11.7]	681
Burundi	36.4 [33.4,39.6]	23.6 [20.9,26.5]	23.2 [20.6,26.1]	1010
Uganda	32.5 [29.6,35.6]	23.9 [21.5,26.4]	18.3 [16.1,20.7]	1675
Tanzania	32.4 [29.2,35.7]	28.9 [25.9,32.0]	11.6 [9.7,13.8]	1491
Zambia	31.8 [29.0,34.8]	26.2 [23.5,29.0]	14.9 [13.0,17.0]	1694
Angola	30.6 [27.2,34.2]	28.5 [25.3,31.9]	9.8 [8.1,11.8]	2086
Namibia	29.1 [20.9,38.9]	26.7 [19.2,36.0]	10.1 [5.5,17.9]	125
Zimbabwe	28.4 [25.2,31.9]	21.5 [18.7,24.6]	12.9 [10.7,15.5]	1081
Malawi	26.1 [23.3,29.1]	15.8 [13.6,18.4]	16.9 [14.5,19.6]	1265
Côte d'Ivoire	25.3 [22.2,28.8]	24.4 [21.3,27.7]	5.9 [4.2,8.1]	960
Kenya	25.2 [21.1,29.8]	21.1 [17.2,25.6]	9.1 [6.5,12.6]	676
Mali	24.7 [20.6,29.2]	20.3 [17.0,24.1]	12.9 [10.3,16.1]	847
Rwanda	23.9 [18.9,29.8]	19.1 [14.7,24.4]	8.4 [5.5,12.8]	219
Ethiopia	21.7 [17.8,26.1]	18.7 [14.9,23.1]	8.4 [5.8,12.0]	814
Chad	18.2 [14.9,22.2]	14.2 [11.5,17.3]	8.7 [6.4,11.9]	948
Nigeria	17.0 [14.8,19.4]	12.8 [10.9,14.9]	8.4 [6.7,10.4]	1530
South Africa	15.8 [9.8,24.5]	12.2 [7.2,19.8]	8.0 [3.6,16.8]	185
Togo	15.1 [12.5,18.0]	11.9 [9.6,14.5]	7.1 [5.3,9.4]	785
Benin	14.2 [11.5,17.3]	9.1 [7.0,11.7]	7.7 [5.7,10.3]	832
Senegal	14.1 [10.3,19.0]	10.4 [6.9,15.2]	8.2 [5.4,12.2]	504
Mozambique	12.4 [9.6,16.0]	11.8 [9.0,15.3]	2.1 [1.2,3.5]	661
Burkina Faso	8.4 [7.1,9.9]	7.8 [6.6,9.1]	1.5 [1.0,2.2]	2475
Gambia	7.1 [5.2,9.6]	5.3 [3.7,7.4]	2.2 [1.2,4.0]	869
Comoros	6.5 [4.4,9.6]	5.2 [3.5,7.7]	2.0 [1.0,4.1]	479

The prevalence of physical IPV varied from 5.2% in Comoros (2012) to 38.8% in Gabon (2012). The prevalence of physical IPV was higher in the Central Africa region where AYW in three of the five countries (Congo DRC, Cameroon and Gabon) reported a prevalence of over 30%. The reported prevalence of sexual IPV ranged from 1.5% in Burkina Faso (2010) to 23.9% in DRC Congo (2013). The prevalence of sexual IPV was highest in Central Africa (16.1%), followed by Southern Africa (10.4%), Eastern Africa (10.1%) and West Africa (7.7%) respectively (Fig. 1). Outside Central Africa (DRC Congo, Gabon and Cameroon) more than one sixth of AYW in countries such as Burundi, Uganda and Malawi reported sexual violence (Table 2).

There is a high correlation between the prevalence of sexual IPV and physical IPV in SSA (Pearson’s correlation, coefficient, r = 0.66), and several countries with high physical IPV (DRC, Gabon, Burundi, Uganda) also reported high levels of sexual IPV. In a few countries, for instance in Burundi and Malawi, AYW reported nearly equal or higher level of sexual IPV than physical IPV. On the other hand, there are a few countries with very low sexual IPV overall; AYW in Burkina Faso, Comoros, Mozambique and Gambia reported sexual IPV prevalence of about 2% or less (Table 2).

**Fig. 2:**
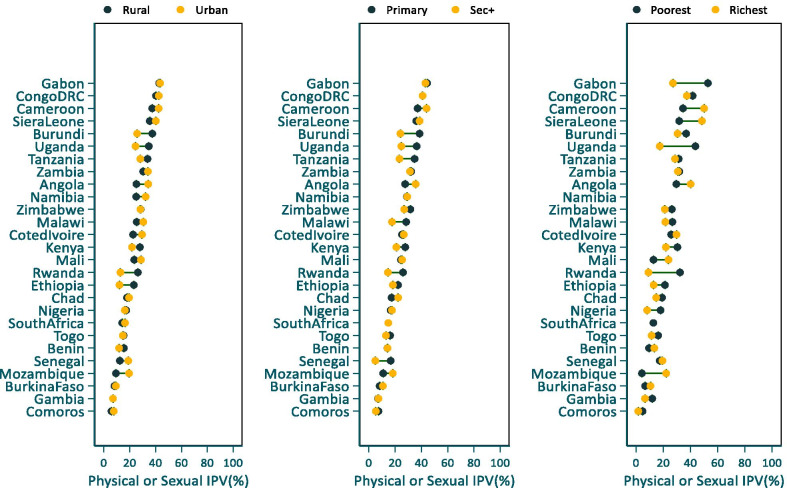
Percentages of AYW reporting physical or sexual intimate partner violence by stratifiers, SSA

A close examination of the prevalence of IPV by the different stratifiers showed no clear overall pattern and little within-country variations in AYW’s experience of physical or sexual IPV in the 12 months preceding the survey (see Additional file 1: Table S1). For instance, there is no clear pattern in rural–urban inequalities in the prevalence of physical or sexual IPV. In the majority of countries (15 of the 27) the prevalence was higher in urban areas although the differences were statistically significant in only three of the 17 countries; Mozambique, Angola, and Cote d’Ivoire (Fig. 2).

In 11 of the 27 countries, a higher proportion of AYW from rural areas experienced physical or sexual IPV in the past year with differences being statistically significant in three countries (Burundi, Ethiopia and Uganda). The pattern of inequality by education was similarly mixed. In 14 out of 27 countries, the prevalence of IPV was higher among AYW with primary education, and the differences were significant in four countries (Uganda, Burundi, Tanzania and Malawi). Physical or sexual IPV prevalence was higher among AYW with secondary and above education in 11 countries but the differences were not statistically significant except in Mozambique (Additional file 1: Table S1).

The patterns by wealth quintile shows that in about three-fifth of countries, a higher proportion of AYW from the poorest wealth quintile reported physical or sexual IPV although the differences were significant for only four countries (Uganda, Tanzania, Rwanda, and Gabon). In nine countries, the reported prevalence of physical or sexual IPV was higher among AYW from the richest wealth quintile. However, the difference was statistically significant in four countries; Mozambique, Senegal, Sierra Leone, and Cameroon (Additional file 1: Table S1).

We also examined inequalities in physical or sexual IPV by AYW’s attitudes towards wife beating, age difference between the partner and AYW’s age. Overall, the prevalence of IPV was higher among young women than adolescents, although the differences were significant in only four countries (Nigeria, Tanzania, Angola and Burkina Faso). In the four countries (Zimbabwe, Malawi, Benin and Senegal) where a greater proportion of adolescents than young women reported IPV, the differences were not statistically significant.

**Fig. 3:**
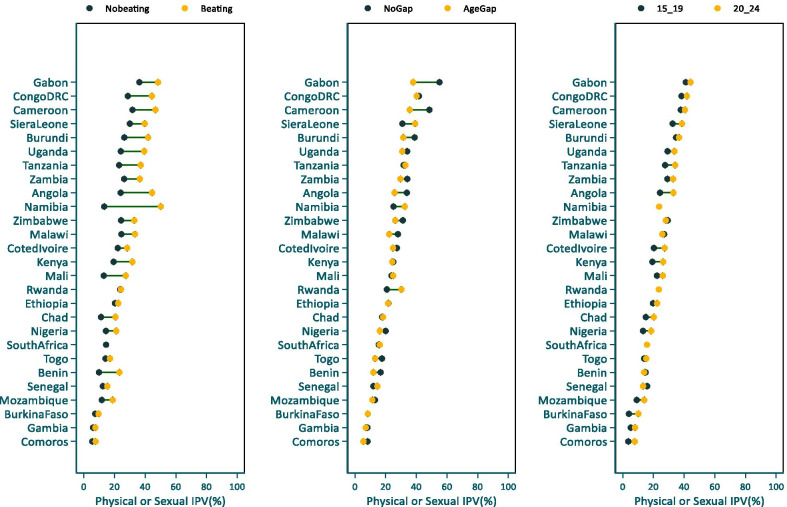
Percentages of AYW reporting physical or sexual IPV by empowerment measures, SSA

The prevalence of physical or sexual IPV varied markedly by attitudes towards wife beating. As shown in Fig. 3, a higher proportion of AYW who approved wife-beating for any reason reported physical or sexual IPV than their counterparts who disapproved wife beating for any reason. The differences were significant at the 5% level in 15 of the 27 countries and remarkable disparities were seen in countries such as Namibia, Mali, Angola, Burundi and Uganda. In Namibia for instance, the proportion of AYW who reported physical or sexual IPV varied from 13.3% among those who disapproved wife-beating to 50.2% among those who accepted wife-beating. The prevalence of physical or sexual violence did not vary significantly by the age difference between the respondent and her partner. However, in many countries, IPV prevalence was higher among AYW whose age difference with the partner was less than 5 years and the differences were statistically Significant in three countries; Cameroon, Burundi, Angola (Additional file 1: Table S1).

### Societal characteristics and reporting IPV

**Fig. 4:**
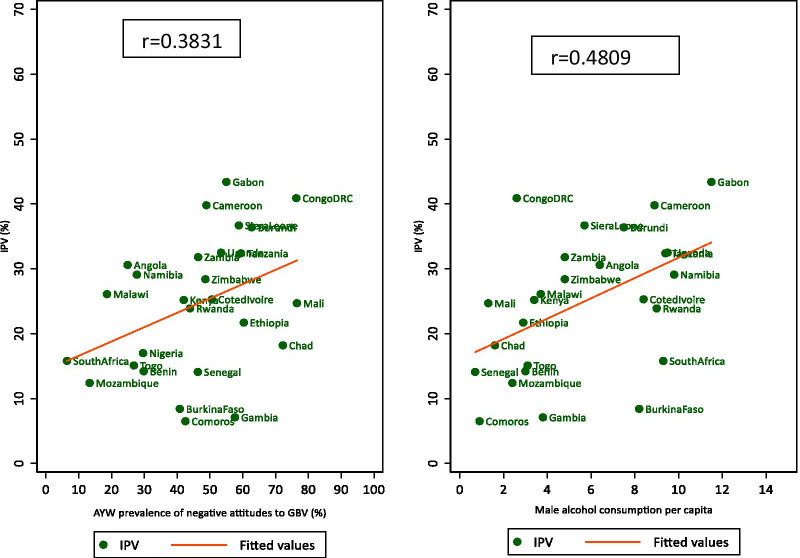
Scatterplot matrix of the correlation between the societal determinant and IPV

While there were observable variations between countries, the within countries variation by the stratifiers was relatively inconsistent. Key questions emerging from this observation are: what explains the large variation between countries? We examined the correlation between reporting IPV at the country level and selected societal characteristics—Gender Inequality Index (GII), educational attainment (proportion of AYW with secondary and above education), GNP per capita, urbanization levels, adult male alcohol consumption per capita and the prevalence of negative attitudes towards wife beating. All bivariate analyses showed no or weak associations between the societal characteristics and IPV among AYW. Moderate or weak correlations were observed with adult male alcohol consumption per capita (Pearson correlation coefficient, r = 0.48) and the prevalence of negative attitudes towards wife beating (Pearson correlation coefficient, r = 0.38). There was no or very weak association with the GII, GNP per capita, Fragile States Index, and level of education of young people in the society (see Fig. 4 and Additional file 2: Table S2).

### Attitude towards wife beating

**Fig. 5:**
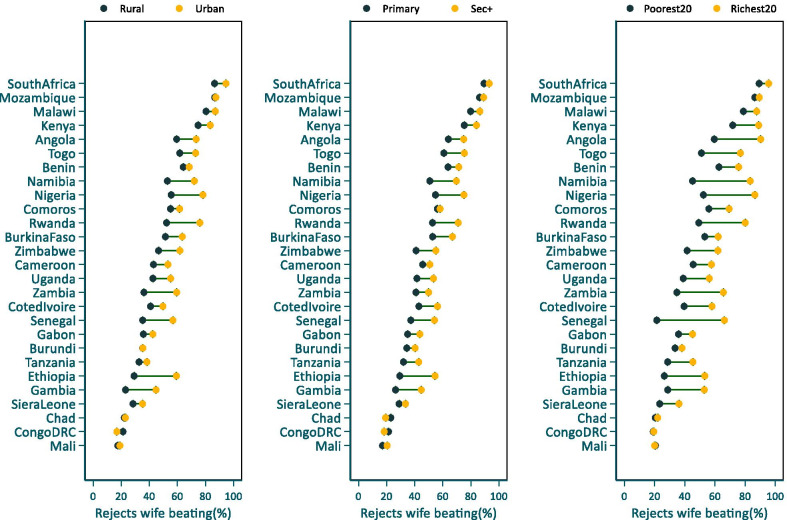
Percentage of adolescents and young adult women who reject wife-beating by stratifiers

The median proportion of AYW who rejected all reasons of wife-beating ranged from 17.9% in Mali (2018) to 92.5% (2016) in South Africa with a median of 47.7% in the 27 countries in the analysis. Moreover, wide within-country inequalities in attitudes towards wife-beating existed by place of residence, wealth quintile and education. In almost all the 27 countries, a higher proportion of AYW residing in urban areas rejected wife-beating compared to their rural counterparts and the differences were statistically significant for 18 countries. Wider rural–urban differences of over 20 or more percentage points were observed in countries such as Ethiopia, Nigeria, Gambia and Namibia (Fig. 5, Additional file 3: Table S3).

The disparity by wealth quintile was also remarkable. In 17 of the 27 countries, the proportion of AYW who rejected wife-beating was significantly higher among the richest quintile compared to the poorest. Large disparities by wealth were observed in Ghana, Nigeria, Angola, Ethiopia, Namibia, Zambia and Senegal. Inequalities by education were also notable with a higher proportion of AYW with secondary education rejecting wife-beating. The differences between AYW with secondary education and those with primary or lower education were statistically significant for 17 countries (see Additional file 3: Table S3).

## Discussion

Findings from these analyses indicate that IPV against AYW is a pervasive problem in SSA with large between and within-country variations. Across the 27 countries included in our analyses, more than one in four AYW reported physical or sexual IPV in the 12 months before the surveys. National prevalence of physical or sexual IPV varied from 6.5% in Comoros to 43.3% in Gabon. Overall, the prevalence of physical or sexual IPV was higher in Central Africa region compared to other sub-regions. In countries such as Gabon, Cameroon, Sierra Leone and Congo DRC over one-third of AYW reported experiencing physical or sexual IPV in the past year. Many of these countries are in conflict or post conflict situations that might have exacerbated pre-existing patterns of violence against women and girls as conflicts can result in higher levels of violence against women and girls, including arbitrary killings, torture, sexual violence and forced marriage [35].

Previous studies show that differences in contextual and structural factors may explain some of the differences observed in IPV prevalence between countries [12, 20, 23]. Our analysis, however, showed that despite observable regional and between-country variation, the reporting of IPV by AYW was not strongly correlated with societal characteristics such as the Gender Inequality Index, GNP per capita, Fragile States Index, or aggregate levels of AYW educational attainment at the national level. It was moderately correlated with male adult alcohol consumption per capita and approval of wife beating at the national level. The poor correlation with Gender Inequality Index is unexpected. Some studies have reported a moderate correlation between the index and IPV among women aged 15–49 in low and middle income countries [36, 37]. Our study shows that conventional indicators of socio-economic development may not explain large inter-country differences in reported IPV against AYW in the region (Fig. 5).

While the between countries variation in the prevalence of IPV is large, the analysis demonstrated that the within country inequalities by the stratifiers are not consistent. However, in close to half of the countries, AYW residing in rural areas, with lower education and those from the poorest wealth quintile experienced more IPV than their counter-parts from urban areas, with higher education and those from the richest wealth quintiles, respectively. In particular, in many countries with a very high prevalence of IPV (Gabon, DRC Congo, Uganda, Burundi and others) a higher proportion of AYW from the poorest wealth quintile experienced IPV compared to their counterparts. Previous studies have highlighted associations between low education, poverty and violence among young women and noted that young women from poor households may have low decision-making abilities, resources and empowerment, which increases their vulnerability to violence [12, 38]. Thus, interventions that broaden women’s access to economic resources and opportunities may help empower women and help reduce the risk of IPV.

The finding that in countries like Angola, Cote d'ivoire, Mali, Mozambique and Burkina Faso, more educated, urban and wealthier AYW were more likely to report IPV is noteworthy. These AYW may challenge the traditional status quo and may be considered more “empowered”. Their male partners may therefore resort to using violence to maintain a dominant position in contexts where male dominance is normative [8, 15]. “Empowered” AYW may also be more willing to disclose IPV [39]. However, these inconsistent patterns also suggest that violence against women and girls is pervasive across all socio-economic backgrounds [3, 20, 23].

While the regional differences in reporting may reflect important cultural, political, or religious differences [40, 41] differential reporting by women of different socio-economic groups within a country is also possible depending on cultural and social norms that underlie the acceptance of violence in each settings [37, 40]. The culture of silence that affects the reporting and what constitutes violence varies across cultures and can make comparability difficult. Evidences show that levels of IPV may be under reported due to fear of retaliation by partners, shame and stigma, lack of awareness of available services or access to such services among other reasons [2, 40]. Interestingly, as approval of wife beating is associated with IPV prevalence, it is possible that AYW who accept wife beating are more willing to disclose experience of IPV than their counterparts who disapprove wife beating. However, while the magnitude of underreporting is unknown, the DHS violence against women module uses validated and standardized questions that are implemented following WHO recommendations of studies on violence against women and girls to improve data quality, protect the safety of respondents and enable comparability across countries.

The ecological model, which is most widely used for understanding the causes of violence, proposes that violence is a result of factors operating at various levels. While there is limited research on community and societal influences, many of the factors identified are context specific and vary among and within countries [14, 42]. The disaggregated analysis showed greater inequality in IPV by AYW’s attitudes towards wife beating, with lower IPV levels among those who reject wife beating for any reason compared to those who accept wife beating. In most countries, trend analysis of DHS data shows that attitudes towards wife beating are changing. However, we found that approval of wife beating is more common among rural dwellers, those from poor households, and those with lower levels of education. Similar findings were recently reported in a publication describing the most vulnerable groups in low and middle-income countries that high IPV prevalence among those who accept wife beating [37]. As documented by several studies [15, 16] social norms play a significant role with a large proportion of AYW from the poorest households viewing spousal violence as a normal and justified occurrence in marriage. Traditional beliefs that men have a right to control women and that increase vulnerability to violence are more common among the poorest, less educated and rural adolescents in SSA [9, 20, 37, 43]. Consistent with the findings of our ecological analysis, the association between partner alcohol use and increased risk of IPV victimization at the individual level has been reported by various studies [44, 45]. These findings have implications for the targeting of violence prevention interventions aimed at promoting more equitable gender norms.

Overall, the findings demonstrate that IPV is pervasive among AYW, with substantial variation between countries and regions reflecting the role of contextual and structural factors in shaping vulnerability to IPV. The lack of consistent pattern of inequalities by the stratifiers within countries shows that IPV against women and girls cuts across socio-economic boundaries suggesting the need for comprehensive and multi-sectoral approaches to preventing and responding to IPV in line with the ecological framework [46, 47]. Moreover, the observed variation by attitudes towards wife-beating shows that promoting gender equitable norms from early childhood through multi-sectoral strategies (including school-based interventions that address gender norms and attitudes from younger ages, and community interventions that can empower women such as microfinance schemes; and media interventions to increase public awareness) can help in reducing violence against women and girls [9, 20, 48]. However, the effectiveness of IPV mitigation actions and care services in SSA remain to be evaluated.

Globally, efforts to prevent and respond to cases of violence against women and girls have increased in the last few decades. Many countries in SSA have adopted laws and policies addressing different forms of violence, including rape, child sexual abuse, and domestic and/or intimate partner violence. However, the implementation of these laws is hampered by weak institutional capacities and limited reporting by victims of violence [30, 48]. Nonetheless, there is an increasing availability of data and lessons from programmatic responses that can be used to scale up prevention and response mechanisms to attain the SDG goal of eliminating all forms of violence against women and girls by 2030.

This study is not without limitations. It is important to note that rates of IPV may be under-reported due to cultural and social norms that underlie the acceptance of violence. Although the DHS violence against women module is implemented following WHO recommendations of research, the fact that the module is implemented within a wide range of health modules means that women are likely to under report due to social desirability bias [49]. Evidence from countries with two or more surveys with the module (e.g., Nigeria, Malawi, Kenya and Rwanda) also shows that prevalence of IPV against AYW between consecutive surveys were not consistent indicative of reporting issues. Moreover, as these modules are implemented in a sub-sample of households and individuals, the sample of married adolescents is relatively small which may have resulted in wide confidence intervals for some parameters. But, we have excluded surveys with less than 30 observations from the analysis.

## Conclusion

Overall, the findings demonstrate that IPV is pervasive among AYW, with substantial variation between countries and regions reflecting the role of contextual and structural factors in shaping vulnerability to IPV. The lack of consistent pattern of inequalities by the stratifiers within countries shows that IPV against AYW cuts across socio-economic boundaries suggesting the need for comprehensive and multi-sectoral approaches to preventing and responding to IPV. The between country variation however is poorly measured by conventional development indicators. On the other hand, the observed variation by attitudes towards wife-beating show that promoting gender equitable norms from early childhood helps in reducing violence against women and girls.

## Supplementary Information

**Additional file 1: Table S1.** Prevalence of IPV among AYW by country and the stratifiers, SSA.

**Additional file 2: Table S2.** Linear regression analysis of the association between selected societal factors and physical or sexual IPV against AYW in SSA>.

**Additional file 3: Table S3.** Attitudes towards wife beating among AYW by country and stratifiers, SSA.

## Data Availability

The dataset used for the current study is available for free from https://dhsprogram.com/data/available-datasets.cfm and can be provided up on request by the corresponding author.

## Abbreviations

AYW: Adolescents and young women
DHS: Demographic and Health Surveys
GBV: Gender based violence
IPV: Intimate partner violence
SDGs: Sustainable Development Goals
SSA: Sub-Saharan Africa
GII: Gender Inequality Index
GNP: Gross National Product
