# Supplementary material for: Intimate partner violence against adolescents and young women in sub-Saharan Africa: who is most vulnerable?
Source: Reprod Health. 2021 Jun 17;18(Suppl 1):119. doi: 10.1186/s12978-021-01077-z (PMC8210343; doi:10.1186/s12978-021-01077-z)
Supplement: Supplementary file 2 — Additional file 2: Table S2. Linear regression analysis of the association between selected societal factors and physical or sexual IPV against AYW in SSA>. [file 12978_2021_1077_MOESM2_ESM.docx]

**Table S2:** Linear regression analysis of the association between selected societal factors and physical or sexual IPV against AYW

| **Variables** | **Unadjusted Coefficients (95% CI)** |
| --- | --- |
| Urbanization levels | 0.155 (-0.096 - 0.406) |
| AYW education attainment | -0.119 (-0.351- 0.112) |
| Attitudes towards wife beating (% accepting) | 0.221 (0.001-.441)* |
| GNI per Capita (log) | 1.067( -3.498- 5.633) |
| Fragile State index (log) | 16.073(-18.081- 50.228) |
| Gender In equality Index | 12.785(-39.89-65.45) |
| Sub-region (RC=Central) |  |
| Eastern Africa | -10.685 (-22.236- .866) |
| Southern Africa | -9.600(-23.407 - 4.207) |
| Western Africa | -17.508 (-29.242- -5.775)* |
| Adult male alcohol consumption per capita | 1.567 (0.363- 2.770)* |
| *** p<0.001, ** p<0.01, * p<0.05 |  |
